# Drought stress has transgenerational effects on seeds and seedlings in winter oilseed rape (*Brassica napus* L.)

**DOI:** 10.1186/s12870-018-1531-y

**Published:** 2018-11-23

**Authors:** Sarah V. Hatzig, Jan-Niklas Nuppenau, Rod J. Snowdon, Sarah V. Schießl

**Affiliations:** 1 Department of Plant Breeding, Justus Liebig University, Heinrich-Buff-Ring 26-32, 35392 Giessen, Germany; 2 Department of Ecology, Environment and Plant Sciences, 106 91 Stockholm, Sweden

**Keywords:** Canola, Rapeseed, Drought stress, Seed germination, Seedling vigour, Metabolite analysis, Seed quality, Fatty acids, Amino acids, Intergenerational stress memory

## Abstract

**Background:**

Drought stress has a negative effect on both seed yield and seed quality in *Brassica napus* (oilseed rape, canola). Here we show that while drought impairs the maternal plant performance, it also increases the vigour of progeny of stressed maternal plants. We investigated the transgenerational influence of abiotic stress by detailed analysis of yield, seed quality, and seedling performance on a growth-related and metabolic level. Seeds of eight diverse winter oilseed rape genotypes were generated under well-watered and drought stress conditions under controlled-environment conditions in large plant containers.

**Results:**

We found a decrease in seed quality in seeds derived from mother plants that were exposed to drought stress. At the same time, the seeds that developed under stress conditions showed higher seedling vigour compared to non-stressed controls.This effect on seed quality and seedling vigour was found to be independent of maternal plant yield performance.

**Conclusions:**

Drought stress has a positive transgenerational effect on seedling vigour. Three potential causes for stress-induced improvement of seedling vigour are discussed: (1) Heterotic effects caused by a tendency towards a higher outcrossing rate in response to stress; (2) an altered reservoir of seed storage metabolites to which the seedling resorts during early growth, and (3) inter-generational stress memory, formed by stress-induced changes in the epigenome of the seedling.

**Electronic supplementary material:**

The online version of this article (10.1186/s12870-018-1531-y) contains supplementary material, which is available to authorized users.

## Background

Drought stress is one of the most important abiotic factors impairing seed and biomass yield in global agriculture. Forecasts predict an increasing frequency of insufficient precipitation and consequent aridity in many parts of the world [1, 2]. Therefore, mankind must develop sustainable strategies to protect crop production as well as crop quality under limiting conditions. Breeding for drought-adapted varieties is an important building block in such a strategy. Modern breeding programs require a profound understanding of the specific implications that water stress has on yield and yield quality parameters. The present study helps expand our knowledge on these implications in winter oilseed rape (*Brassica napus* L.), by providing data on maternal and transgenerational effects of drought stress on yield, seeds and seedling vigour. *B. napus* is one of the most important oil crops worldwide [3].

Elimination of seed erucic acid and reduction of seed glucosinolate content (double-low seed quality) facilitated a global boom in production of oilseed rape and canola (*B. napus*), today the second-most important oilseed crop in the world behind soybean. Besides its use as feedstock in Europe and for biofuel production, the oil from oilseed rape and canola also plays a significant role for human consumption. Due to its favourable fatty acid composition with high amounts of mono- and polyunsaturated fatty acids, the consumption of oilseed rape and canola oil has been described to benefit human health [4]. Although desirable seed oil qualities are genetically fixed in modern germplasm collections, moderate fluctuations in seed quality and composition can be linked to environmental influences prevailing during seed production [5–8]. Besides the need for high oil yield, high amounts of desirable fatty acids and low concentrations of undesirable seed components, such as erucic acid and glucosinolates, a fast and uniform germination and high seed vigour are essential for good crop establishment and yield stability. Moreover, enhanced seed and seedling vigour improve plant density and spatial arrangement in the field, extend the growth duration [9] and have a direct positive effect on crop formation and growth [10]. The need for optimal seed germination and vigour characteristics thus requires seed production environments that maximise vigour performance.

Here, we analysed the effect of limiting water conditions during seed production in two respects: First, we investigated the consequences of maternal drought stress on seed yield and seed quality. Subsequently, we evaluated the effect of maternal water supply on germination and seedling vigour performance of progeny from stressed and non-stressed maternal plants of genotypes with varying maternal response to drought stress. Plants from eight contrasting winter oilseed rape genotypes were grown under optimal water supply vs. water shortage during the critical phase of flowering, in a large-container growth system which accurately simulates a field growth environment with deep soil whilst allowing careful control of water supply under semi-controlled greenhouse conditions [11]. As expected, optimal water supply during seed production ensured high seed quality and composition. Unexpectedly, however, lower quality seeds from plants grown under drought stress showed favourable effects on seedling vigour potential in comparison to the seeds from non-stressed plants. This indicates that the optimal growth environment in respect to water availability may differ for farmers and seed producers. While drought can pose a significant threat to seed quality and yield for farmers, drought stress effects during seed production may be advantageous for seed producers in terms of optimising germination and vigour characteristics of commodity seed.

## Results

### Maternal treatments: Seed yield and seed quality

**Fig. 1:**
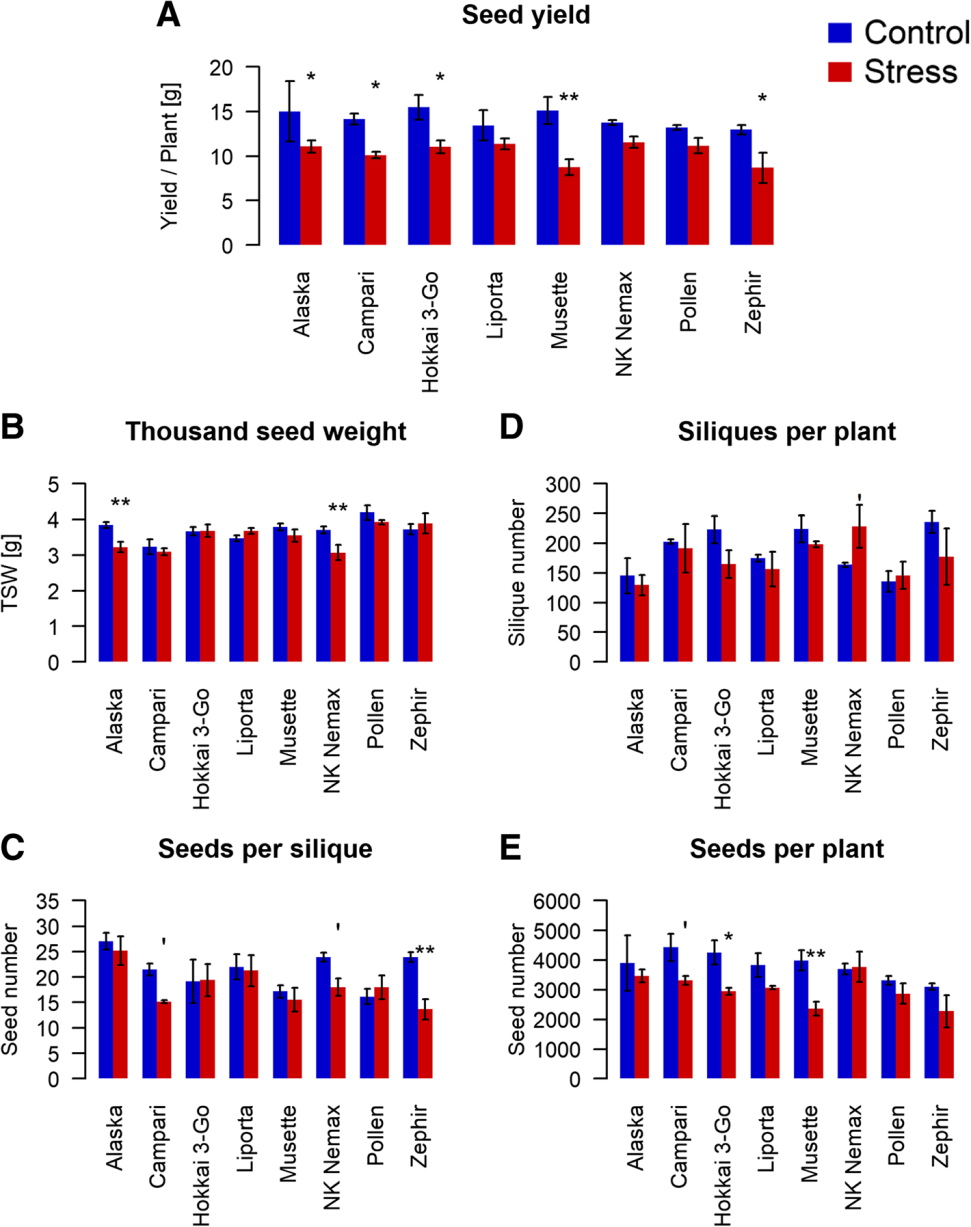
Effect of drought stress on (**a**) Total seed yield, (**b**) Thousand seed weight, (**c**) Number of seeds per silique, (**d**) Number of siliques per plant and (**e**) number of seeds per plant of 8 diverse winter oilseed rape genotypes cultivated in a semi-controlled container trial. Bars are means of three replicates with standard errors. Significant differences at *p* < 0.1′, *p* < 0.05* and *p* < 0.01**

The maternal plants were subjected to a 3 weeks water withdrawal starting shortly before flowering. Total seed yield was significantly affected by this drought treatment for all accessions except *Liporta*, *NK Nemax* and *Pollen* (Fig. 1a). The strongest yield reduction under stress (42%) was found in the accession *Musette*. For accessions *Alaska*, *Campari*, *Hokkai 3-Go* and *Zephir*, we observed reductions of 26, 29, 29 and 33%, respectively. Significant reduction in thousand seed weight was observed only for *Alaska* and *NK Nemax* (Fig. 1b)*.* The number of seeds per silique was significantly reduced in *Campari*, *NK Nemax* and *Zephir* under drought stress (Fig. 1c), however no significant change in number of siliques per plant was observed except in *NK Nemax*, which showed a significant increase in number of siliques under drought stress (Fig. 1d). Number of seeds per plant was significantly reduced in genotypes *Campari*, *Hokkai 3-Go* and *Musette* (Fig. 1e).

**Fig. 2:**
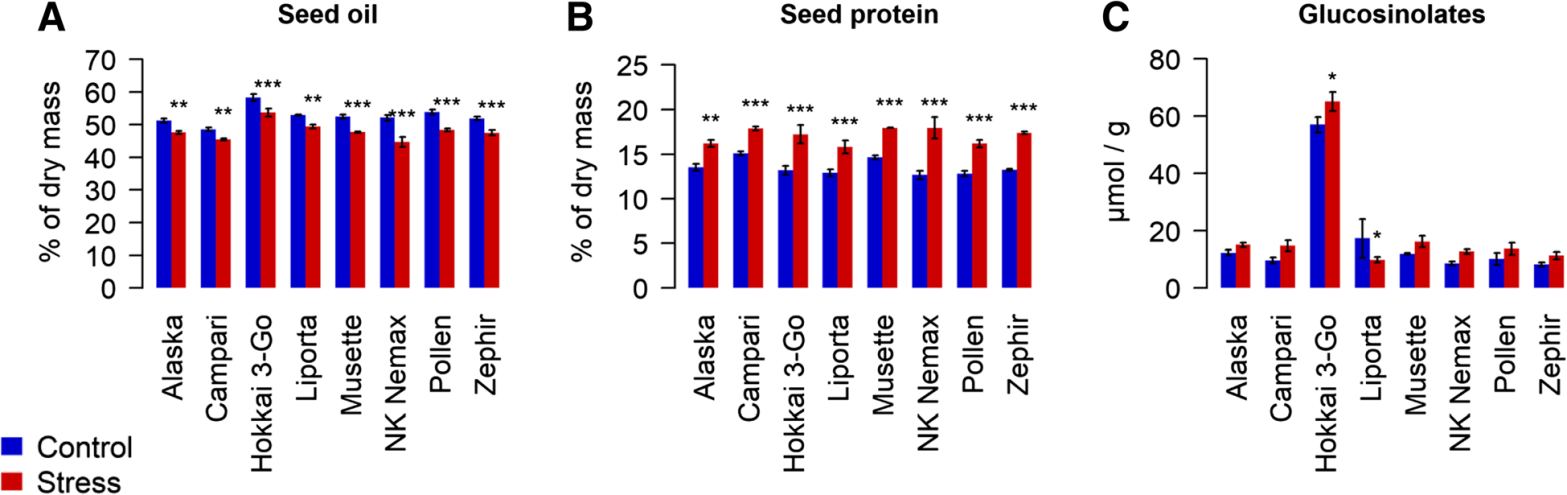
Effect of drought stress on (**a**) seed oil amount, (**b**) seed protein amount and (**c**) concentrations of seed glucosinolates of 8 diverse winter oilseed rape genotypes cultivated in a semi-controlled container trial. Bars are means of three replicates with standard errors. Significant differences at p < 0.05*, p < 0.01** and at *p* < 0.001***

Seed oil and protein content along with the fatty acid composition were strongly affected by the drought stress treatment (Fig. 2). Under drought treatment all accessions reacted with a significant decrease in seed oil content (Fig. 2a) and corresponding significant increase in protein content (Fig. 2b). The strongest effect was observed in *NK Nemax*, which showed 14.4% reduced seed oil and 29.6% increased protein. The weakest effect was seen in *Campari* where oil decreased by 6.5% and protein increased by 15.6% under drought stress. In the drought stress treatment, a general tendency towards higher seed glucosinolate contents was observed, but this effect was statistically significant in *Hokkai 3-Go* only (+ 12.5%; Fig. 2c). In contrast to this trend, *Liporta* showed a significant decrease in seed glucosinolate content (− 42.6%) in the drought stress treatment.

**Fig. 3:**
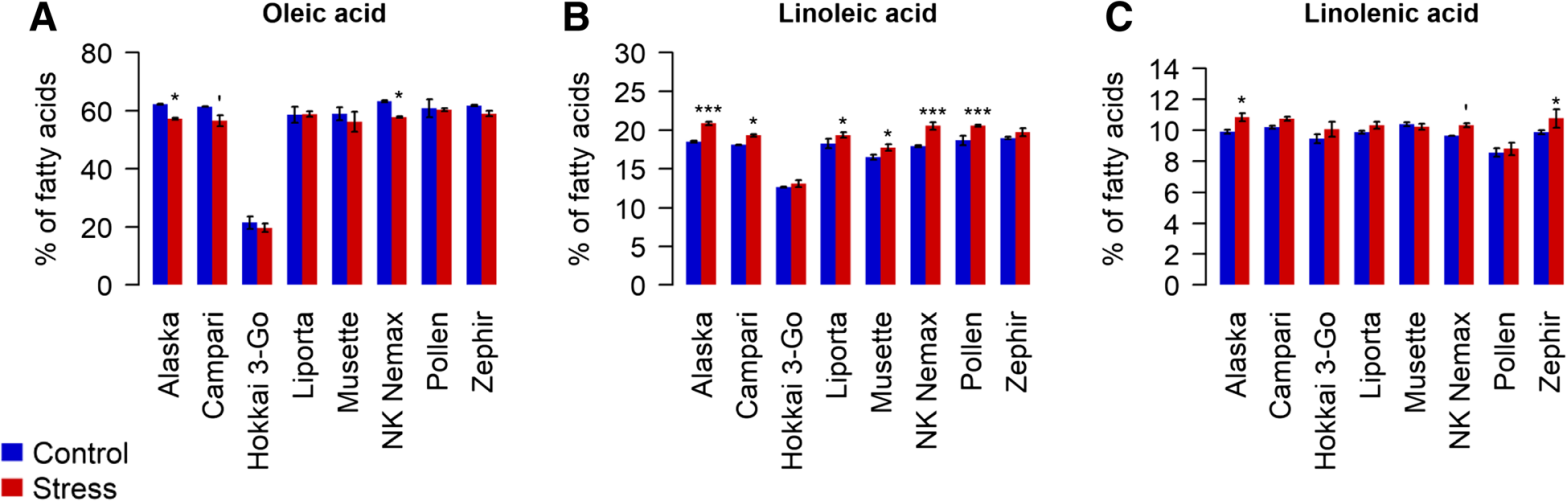
Effect of drought stress on the amount of (**a**) Oleic acid, (**b**) Linoleic acid and (**c**) Linolenic acid measured in the seeds of 8 diverse winter oilseed rape genotypes cultivated in a semi-controlled container trial. Bars are means of three replicates with standard errors. Significant differences at p < 0.1′, p < 0.05* and at p < 0.001***

Seeds produced under drought stress showed distinct changes in fatty acid patterns compared to seeds developed under optimal water conditions (Additional file 1)*.* Oleic acid was significantly decreased in the genotypes *Alaska*, *Campari* and *NK Nemax,* while mean decreases for *Hokkai* 3-Go, *Musette* and *Zephir* were not statistically significant (Fig. 3a). All genotypes except *Hokkai 3-Go* and *Zephir* showed a significant increase in their proportion of linoleic acid (Fig. 3b), while significant increases in linolenic acid were observed for *Alaska*, *NK Nemax* and *Zephir* (Fig. 3c). Other fatty acids (stearic acid, linoleic acid, linolenic acid, palmitic acid, gadoleic acid, behenic acid, palmitoleic acid, lignoceric acid and myristic acid) were generally elevated in the maternal drought variant in three or more of the eight accessions. No effects of drought stress were observed for eicosenic or erucic acid.

### Seed germination characteristics

The absolute germination rate (GR96) under constant watering (0 Mpa) showed a small but significant increase in seeds derived from the maternal drought treatment in *Liporta*, *Musette*, *NK Nemax* and *Zephir*, which showed GR96 values of 99, 99, 96 and 98% in seeds from the maternal control treatment but germinated to 100% in seeds from the maternal drought treatment. *Alaska*, *Campari*, *Hokkai 3-Go* and *Pollen* showed 100% germination in both maternal treatments. Under moderate osmotic stress (− 0.5 Mpa), no significant difference in GR96 was be observed between the maternal treatments, with mean GR96 values of 98.3 and 97.5% for maternal control and drought treatment, respectively.

**Fig. 4:**
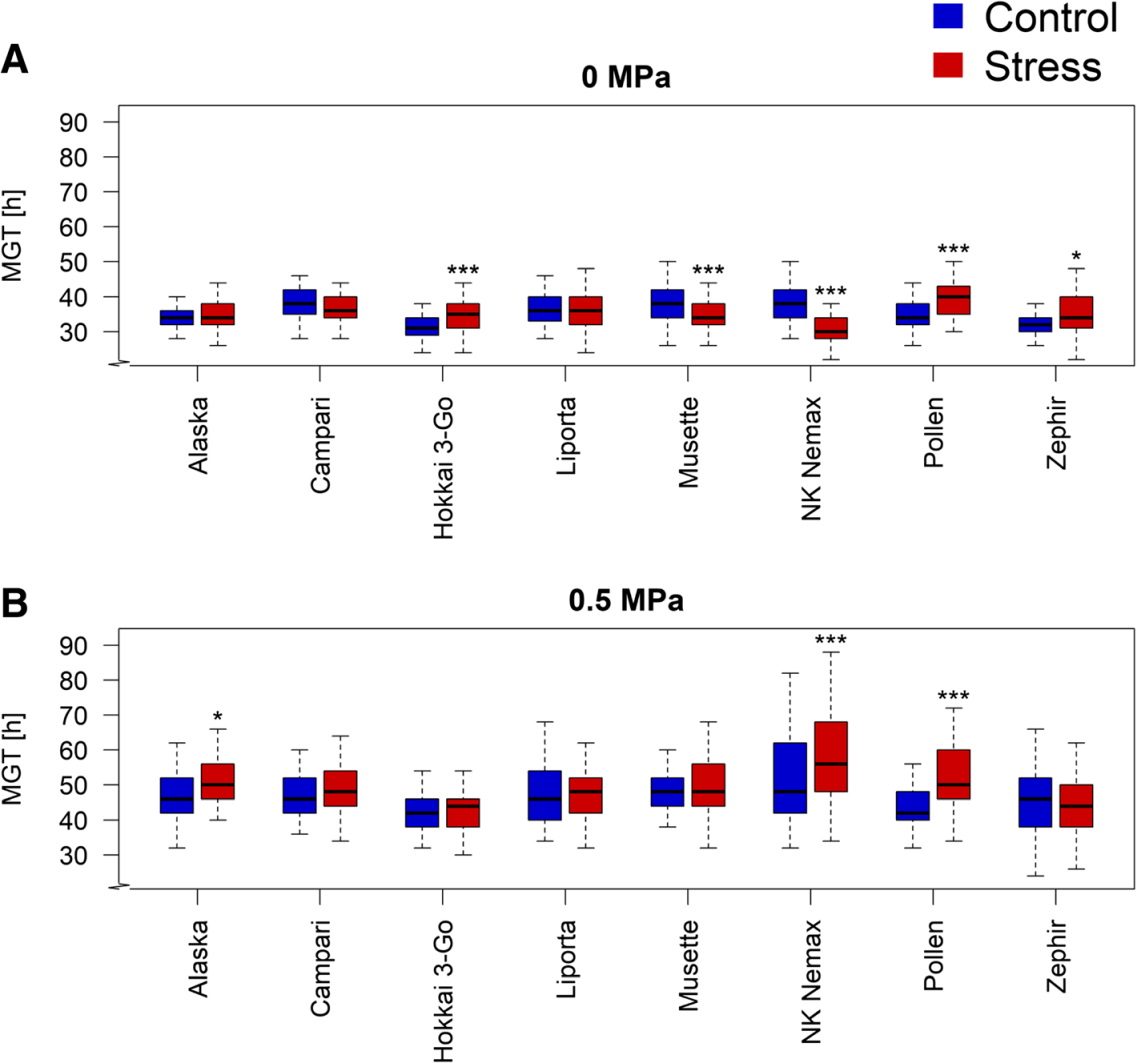
Effect of maternal drought stress on mean germination time (MGT) of seeds from 8 diverse winter oilseed rape genotypes, cultivated in a semi-controlled container trial: (**a**) germination performance under 0 MPa and (**b**) germination performance under moderate osmotic stress (− 0.5 MPa). Boxplots represent performances of 100 seeds each. Significant differences at p < 0.05* and at p < 0.001***

Accessions reacted significantly different in their mean germination time (MGT) under constant watering (Fig. 4a). Whereas *Hokkai 3-Go*, *Pollen* and *Zephir* showed a significant increase in MGT in seeds from the maternal drought treatment, *Musette* and *NK Nemax* showed higher MGT values in the seeds from the maternal control treatment. MGT was equal for both maternal treatments under moderate osmotic stress, except in *Alaska*, *NK Nemax* and *Pollen*, which showed higher MGT values in the maternal stress treatment (Fig. 4b). Comparing the two in vitro scenarios, constant watering and moderate osmotic stress, uniformity (U) in seed germination was significantly higher under constant watering (data not shown). However, no significant difference in U was observed between the two maternal treatments, neither under constant watering nor under moderate osmotic stress.

### Seedling vigour performance

**Fig. 5:**
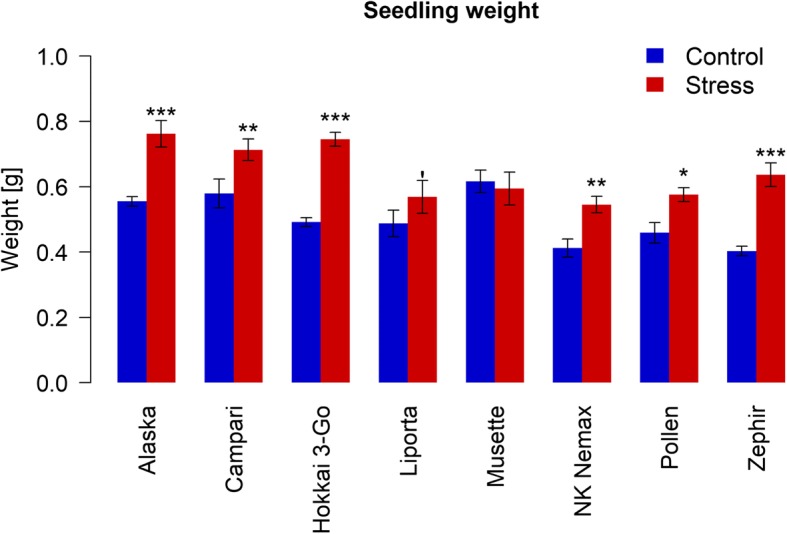
Effect of maternal drought stress on seedling growth performance of seeds harvested from 8 diverse winter oilseed rape genotypes cultivated in a semi-controlled container trial. Bars are means of three replicates with standard errors. Significant differences at p < 0.1′, p < 0.05*, p < 0.01** and p < 0.001***

Significant differences in seedling vigour were observed both between accessions and the two different maternal water treatments (Fig. 5). For all accessions except *Musette*, seedling fresh weights were found to be significantly increased by the maternal drought stress treatment. The strongest effect was found in *Zephir* which showed a mean increase of 36.7% relative to the control treatment, followed by *Hokkai 3-Go* and *Alaska* with 34.0 and 27.2% increase, respectively. The lowest significant difference was observed for *Liporta,* which showed an increase by 14.3% relative to the control. Seedling vigour was found to be uncorrelated to mean germination time for both maternal water treatments. A replication trial with 4 of those 8 accessions in the next season showed the same trend (Additional file 2: Figure S5).

### Seedling metabolite patterns

In seedlings from the maternal drought treatment, concentrations of several amino acids and nitrogen compounds were significantly increased in diverse accessions compared to seedlings from the maternal control treatment (Additional file 3). From these metabolites, ammonium (NH_4_^+^), Histidine (His), Asparagine (Asn), S-methylcystein sulfoxide (SMCSO), Glutamine (Gln), Arginine (Arg), Glycine (Gly), Aspartate (Asp), Threonine (Thr), α-Alanine (α-Ala), γ-aminobutyric acid (GABA), Tyrosine (Tyr), Valine (Val) and Isoleucine (Ile) showed significantly higher concentrations in three or more of the eight genotypes under investigation. The concentrations of sugars and organic acids were similarly increased in the maternally drought treated seedlings of several accessions (Additional file 3). Most differences between the maternal treatments were observed for malate, fructose and sucrose (significantly different for six, six and three of the eight accessions, respectively). A significant role in the trans-generational response to drought stress was implicated for NH_4_^+^, SMCSO, Gln, Gly, Tyr, Val, fructose and malate. Strong correlations were found between relative concentrations of these metabolites and relative seedling FW in seeds from the maternal drought treatment compared to the control (Additional file 4, Fig. 6).

**Fig. 6:**
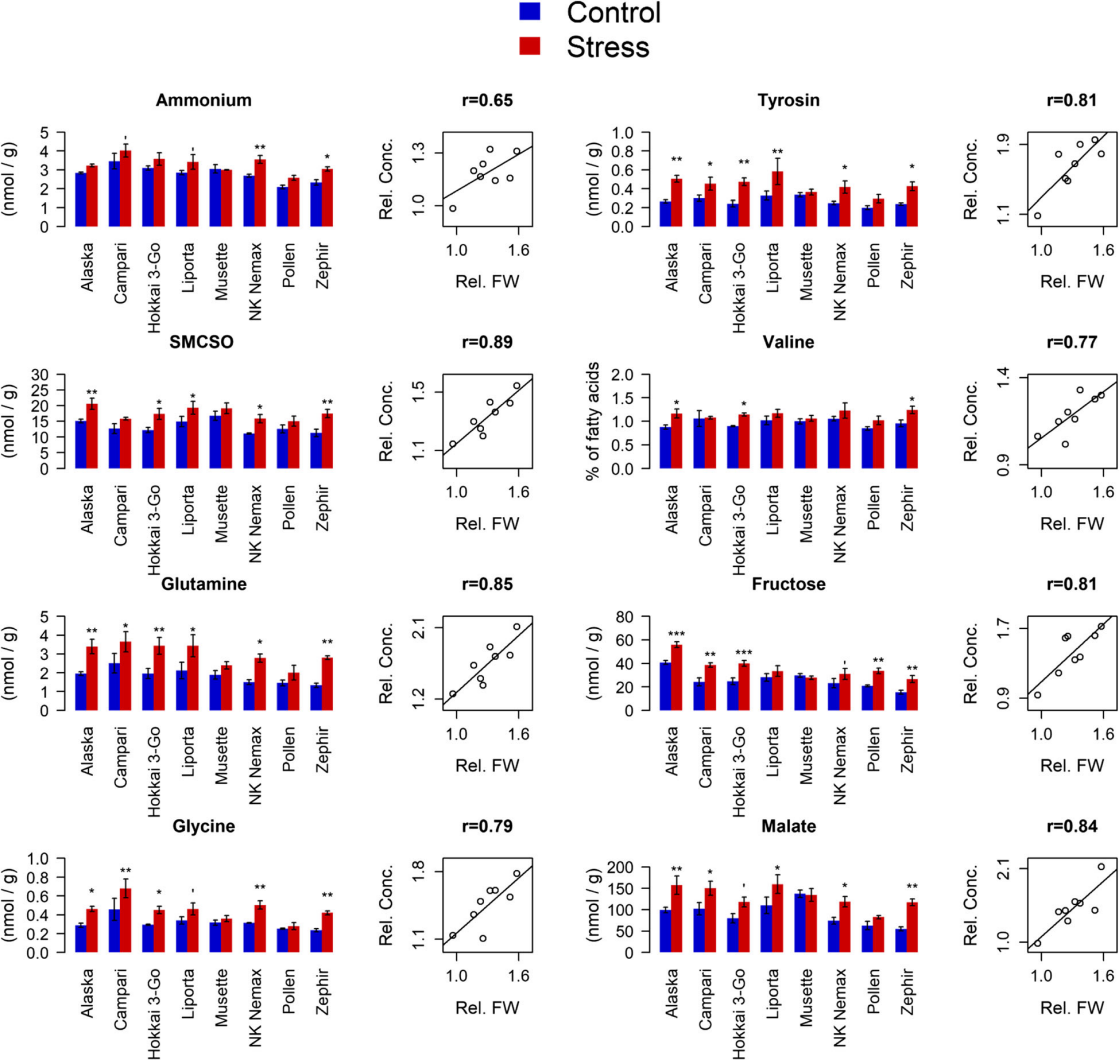
Effect of maternal drought stress on seedling metabolite concentrations of the offspring derived from 8 diverse winter oilseed rape genotypes cultivated in a semi-controlled container trial. Bars are means of three replicates with standard errors. Significant differences at p < 0.1′, p < 0.05*, p < 0.01** and p < 0.001***. Scatter diagrams show correlations between the corresponding relative metabolite concentration (Rel. Conc.) and relative seedling fresh weight (Rel. FW) under drought stress

### Multivariate analysis of seed quality, seedling vigour and seedling metabolic patterns

**Fig. 7:**
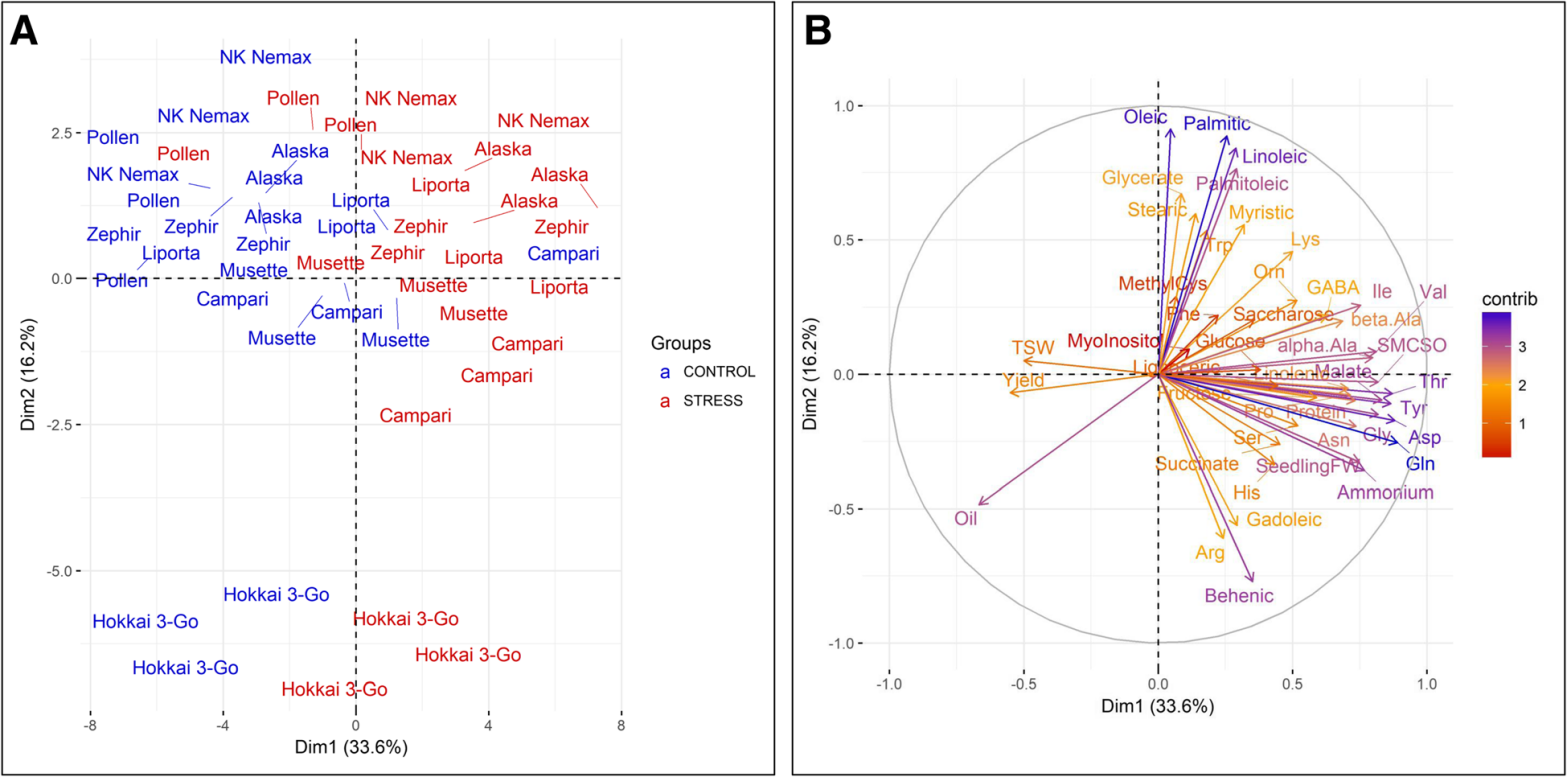
Two-dimensional principal component analysis showing (**a**) the multivariate variation in terms of metabolite concentrations among seeds and seedlings derived from 8 open-pollinated winter oilseed rape genotypes cultivated under control and drought stress conditions in a semi-controlled container trial. All biological replicates are shown. Seedlings from maternal control conditions are shown in blue, seedlings from maternal stress conditions are shown in red. Proximity between samples indicates similarity in metabolite patterns. The axes show the first and second principal component along with the variation explained by them in brackets. (**b**) The same data set now represented as variable vectors indicating the strength and direction of each seed component and seedling metabolite. Vectors which are perpendicular to each other show independence of the component, vectors pointing in opposite directions show a negative correlation, while vectors with a small angle between them show a positive correlation. The color denotes the weight of the contribution to the total variation in the data set, with higher values meaning a stronger contribution. Amino acids are abbreviated as 3 letter IUPAC code. Fatty acids are named without the extension “acid”. TSW: Thousand seed weight, FW: Fresh weight

Using a principal component analysis (PCA) for all seed quality characteristics and seedling metabolite concentrations with significant differences between the maternal treatments, we found a clear spatial separation between offspring plants derived from the maternal stress and maternal control treatment (Fig. 7a). It is evident that the most important determinant is the maternal treatment, while genotypic specificities were subordinated. The accessions *Hokkai 3-Go* showed a similar separation between the maternal treatments but diverged from all other genotypes. As *Hokkai 3-Go* was the only genotype with ++ seed quality, this spatial distance may be explained by differences in its fatty acid patterns. The strongest contributions regarding the separation of quality groups could be attributed to the fatty acids oleic acid, palmitic acid, linoleic acid and palmitoleic acid (Fig. 7b). The largest separating effect would be expected from erucic acid, however this fatty acid was not considered as a variable in the PCA, as it showed no significant differences between the maternal treatments. Furthermore, Fig. 7b shows that Thr, Tyr, malate, Tyr, Asp, Gly, Gln and NH_4_^+^ mainly contribute to the separation between maternal control and stress treatment.

## Discussion

Our data show that during the critical stage of flowering, water supply has a pronounced transgenerational effect on seedling vigour of the progeny. The strength of the drought stress effects on both maternal seed yield and transgenerational seedling performance is strongly genotype-specific. The drought-induced reduction in seed yield confirms the long-known relationship between water supply and yield development during the critical phase of flowering [12–14]. Unexpectedly, the positive transgenerational effect of drought stress on vigour is in contrast to the negative effect of maternal stress on the yield performance of the maternal plants. However, the growth-stimulating transgenerational effect in seedlings due to maternal drought stress was independent from the extent of yield reduction in the mother plant. Interestingly, maternal drought stress had differential effects on seed germination performance depending on the accession. However, the accession had no effect on seedling vigour. In fact, the lack of any significant correlation between Mean Germination time (MGT) and seedling fresh weight suggests that the seedling performance is completely decoupled from germination performance. Our experiments under two different osmotic conditions suggest that the water availability during germination has a stronger influence on germination performance than water availability during foregoing seed production. In contrast, seedling development appears to be clearly enhanced by maternal drought, even in accessions like *Hokkai 3-Go, Pollen and Zephir* which showed delayed germination in seeds of the maternal drought treatment and subsequently exhibited a higher seedling biomass than the maternal control treatment. In a smaller replication trial performed in the following season, the same enhancement effect was observed. These findings contradict the assumption that optimal seedling development presupposes an appropriate germination performance. Instead, our findings corroborate a weak link between germination and post-germination seedling growth, as already hypothesized by [15].

Three possible explanations are proposed for the observed transgenerational effects on seedling vigour: Heterosis, changes in seed quality, and intergenerational stress memory involving an alteration of growth-effective metabolic processes.

In our experiments, we used openly pollinated plants to avoid known negative effects of bagging on seed quality. In consequence, we expect a certain level of cross-pollination, and differences in vigour between progenies from different stress environments could possibly arise from differential outcrossing rates for stressed and control progenies, resulting in different levels of seedling heterosis. Assuming that drought stress negatively affects male fertility [16, 17], higher cross pollination rates might be expected for drought stressed mother plants. Moreover, it has been shown that an enhanced biomass development due to heterosis can already be observed during the early stage of seedling growth [18]. To investigate this phenomenon, we performed a separate quantification of homozygous- and heterozygous progenies by Kompetitive Allele Specific PCR (KASP) genotyping (data not shown), however this analysis did not reveal significant differences in cross pollination rates between the two maternal treatments. Hence, we believe that differential heterosis does not underlie the effects of maternal drought stress on seedling vigour of the progeny.

**Fig. 8:**
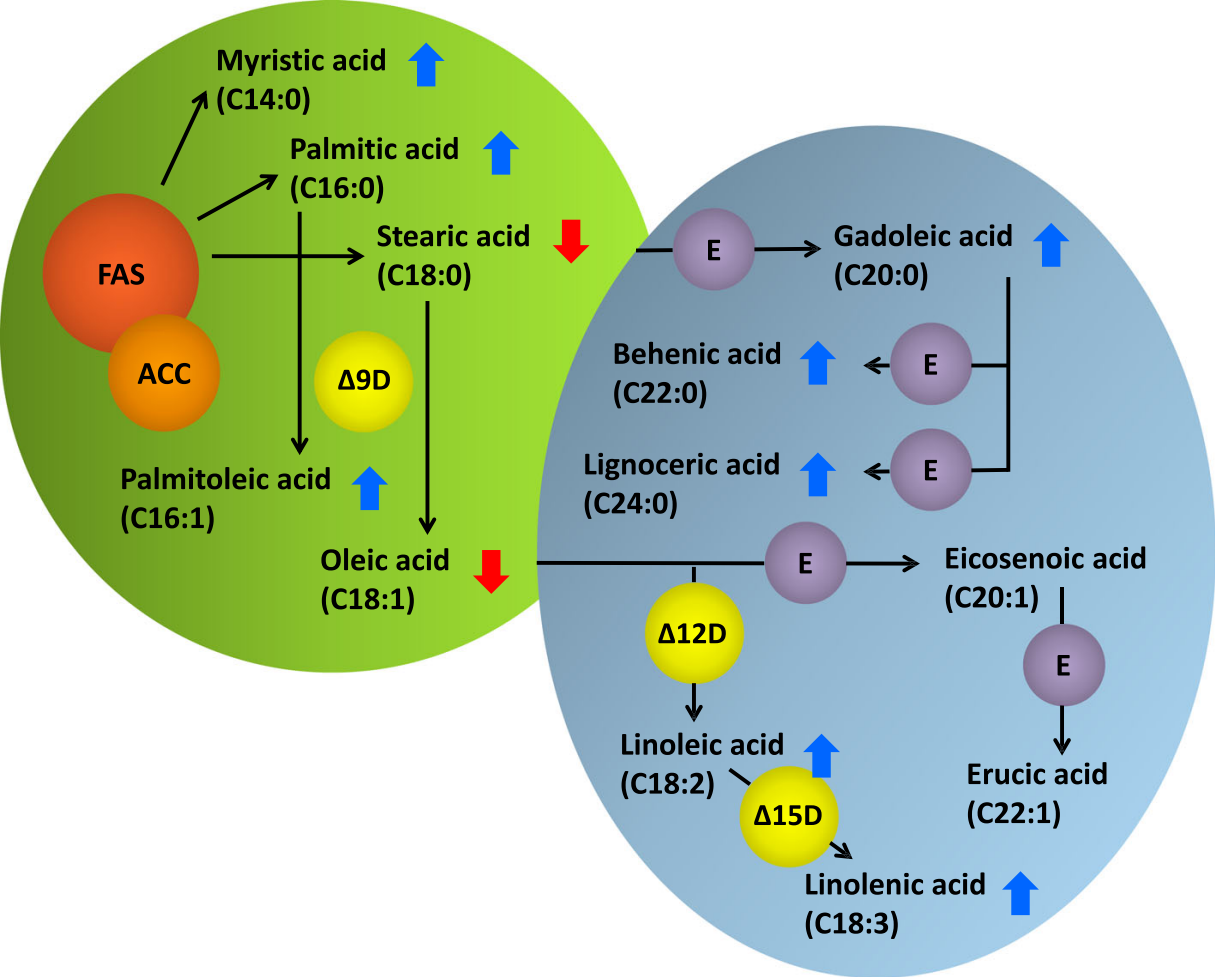
Principal scheme of fatty acid biosynthesis in chloroplasts (green body) and endoplasmatic reticulum (blue body) of rapeseed. Arrows indicate, whether relative amounts of fatty acids have increased or decreased under drought stress in three or more of the 8 observed winter oilseed rape genotypes. FAS: Fatty acid synthase, ACC: Acetyl-CoA-Carboxylase, Δ9D: Δ9-Desaturase, Δ12D: Δ12-Desaturase, Δ15D: Δ15-Desaturase, E: Elongase

Changes in seed quality due to the maternal drought treatment are another possible reason for improved seedling vigour. Changes in seed composition alter the reservoir of storage metabolites on which the seedling relies during post-germination seedling growth. A marked reduction in seed oil content and an associated increase in seed protein content are common observations under drought stress [5, 13, 19, 20]. Considering the strong genotype x environment interaction for seed quality in *B. napus* [21], all genotypes would be expected to react to maternal drought with a similar shift in their seed composition patterns. Increased glucosinolate production in seeds maturing under water stress, as suggested by [5], could not be confirmed statistically by our results. In contrast, we observed a shift in fatty acid patterns, especially at the expense of oleic acid and in favour of polyunsaturated fatty acids like linoleic and linolenic acid, due to reduced water supply [20]. The accessions *Liporta*, *NK Nemax* and *Pollen*, showing no significant yield reduction under stress, nevertheless showed similar declines in seed quality – especially in terms of seed oil content, seed protein content and fatty acid composition. This shows that seed quality is more sensitive to drought stress than the total yield levels. We assume that fatty acid modification processes towards long-chained, poly-unsaturated fatty acids proceed without restriction, whereas the delivery of corresponding precursors like stearic or oleic acid appears to have been limited by the stress (Fig. 8). However, we could not confirm that this led to a net increase in storage lipid biosynthesis beyond the extent given under non-stress conditions. Gene expression analysis has shown that expression of genes encoding fatty acid modifying enzymes like fatty acid elongase 1 (FAE1), one of the core enzymes involved in erucic acid biosynthesis in the *Brassicaceae*, peak to a later time-point than the basic fatty acid synthesis machinery [22]. Therefore, a higher quantity of prolonged and poly-unsaturated fatty acids in the seed might account for an earlier onset of maturity [20]. However, it is unlikely that premature maturity explains the observed shift in fatty acid patterns. Indeed, we found that the flowering period was prolonged under water shortage in our experiment (data not shown), suggesting a delayed maturity.

**Fig. 9:**
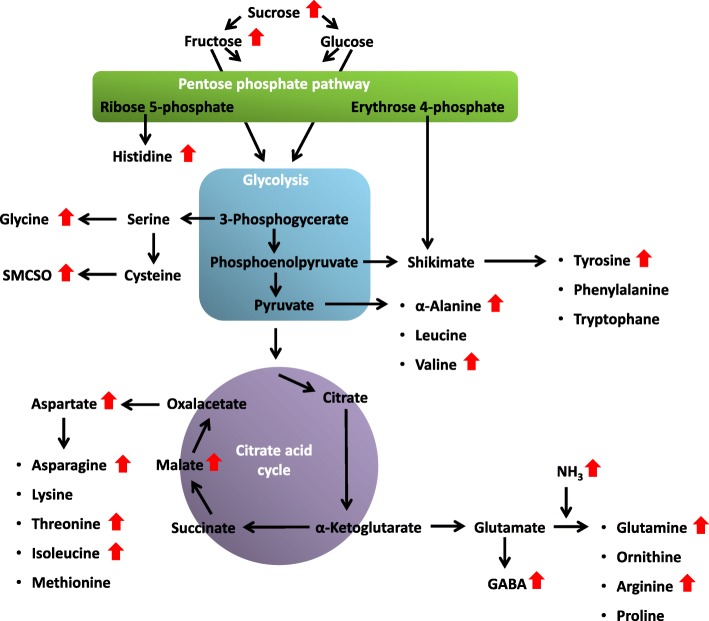
Principal scheme of the biosynthesis and derivation of different amino acid and nitrogen compounds in rapeseed. Arrows indicate, whether concentrations of metabolites have increased or decreased under drought stress in three or more of the 8 observed winter oilseed rape genotypes

Besides seed oil and protein quality parameters, seedling metabolite patterns were also substantially shifted by the maternal drought treatment (Fig. 9). Higher concentrations of free amino acids could be explained by enhanced amino acid biosynthesis and/or enhanced protein degradation or, alternatively, an inhibition of amino acid degradation and/or protein synthesis. Higher monosaccharide- and disaccharide concentrations in seedling tissues might indicate an inhibition of glycolytic processes and/or polysaccharide synthesis, or an enhanced carbon assimilation and/or polysaccharide degradation. As seedling growth is generally enhanced due to maternal drought stress, it appears more likely that growth promoting processes, like carbon assimilation were stimulated, rather than an enhancement of catabolic processes. A possible explanation for an alteration of metabolic processes in the seedling which ultimately lead to enhanced seedling growth is intergenerational stress memory, defined as “a stress imprint that extends from one stressed generation of organisms to at least the first stress-free offspring generation” [23]. This model explains transgenerational effects by stress-induced changes in the epigenome of the plant, amongst them changes in DNA methylation patterns [24, 25] or histone modifications [26]. Such transgenerational effects can instil an adaptive advantage when the progeny is exposed to the maternal stress conditions [24, 27]. While we cannot exclude the possibility of epigenetic changes in our study, we can at least not confirm the adaptive value of the induced changes. However, faster growth before stress induction can improve the plants’ survival chances in a new stress scenario and might therefore represent an adaptation in life cycle. Germination performance in particular showed no advantage in seeds derived from maternal drought treatment under osmotic stress conditions. If such epigenetic changes were present, our data suggest that they are mainly maternally inherited. Otherwise, we would have expected differences in seedling biomass and metabolite patterns among individuals from the same maternal stress treatment, as pollination was equally likely to occur from both stressed or non-stressed pollinators. This is in agreement with other studies suggesting that although both the maternal as well as the paternal environment can form specific transgenerational responses, the post-zygotic maternal effects were generally stronger [28–30].

## Conclusions

The conclusions from our study have opposite consequences for farmers and seed producers. Farmers are primarily interested in achieving high seed yield levels with optimum seed quality, hence drought during seed maturation can have a negative influence. In contrast, commercial seed producers might possibly take advantage of water deficits during the generative growth phase of winter oilseed rape, as seedling vigour performance could be positively influenced by maternal drought stress, for example by generating seeds in water-limited production areas. On the other hand, a commercial benefit can only be achieved if the drought environment does not reduce seed number. This was at least not the case for most of the observed genotypes under the stress conditions applied within our study (Fig. 1e). Seed weight was not negatively correlated to seedling vigour in six out of the eight investigated genotypes in our study, meaning that a reduction in thousand seed weight may not have a negative effect on seed number. In summary, our results provide an interesting new approach for optimization of the commercial seed production process in winter oilseed rape.

## Methods

### Plant material and cultivation of maternal plants

We selected 8 diverse winter oilseed rape (WOSR) inbred lines from the ERANET-ASSYST diversity set [31] based on previous analyses of their flowering time and field performance under water-limiting conditions. The genotype selection comprised inbred lines of the double-low seed quality accessions *Alaska*, *Campari*, *Liporta*, *Musette*, *NK Nemax*, *Pollen* and *Zephir* along with the high erucic acid, high glucosinolate genotype *Hokkai 3-Go*. In the growing period 2014/2015, we grew all accessions under semi-controlled conditions in the large container growth system described by [11], which enables simulated field growth conditions and planting densities with a deep soil profile and exact control of soil water capacity. The containers were filled to a depth of 90 cm with a mixture of 25% soil and 75% sand. Around 30 seeds per container were sown by hand on 30.11.2014. Seedlings were thinned to 9 plants per container 25 d after sowing, with uniform spacing of the nine remaining plants. The plants were fertilized on 02.04.2015 with 1.6 g NH_4_NO_3_ per container. Each genotype was cultivated under well-watered conditions (control) and drought stress conditions (stress). For each accession and treatment, three containers were set up as independent biological replicates, making a total of 48 test containers. The containers were ranged in a fully randomized block design. Additional planted containers were arranged around the container block to minimize boundary effects. Optimal water supply was guaranteed by watering the plants to a minimum soil water capacity of 60%. Water demand was determined by regular weighing of the containers with a hydraulic scale hoist. In the stress treatment water supply was suspended for three weeks from begin of flowering (BBCH 50) until the onset of full flowering (BBCH65). Afterwards the plants were re-watered to the same level as the control treatment (60% water capacity). Soil water capacity was calculated based on container weights assuming a soil dry weight of 138.9 kg, a container weight of 12 kg, and a varying total plant weight. Total plant weight was estimated to be 1 kg at the start of the stress treatment. For control plants, we assumed a biomass gain of 100 g per container and day. For stress plants, we assumed a biomass gain of 100 g per container and day in the first week, 50 g per container and day in the second week, and no gain in the second week. The resulting differences in soil water content before and after watering are shown in Additional file 5: Figure S4. All plants were openly pollinated. Seeds from both the well-watered and drought stress treatment (hereinafter referred to as maternal treatments) were harvested on 13.07.2015 and stored under dry conditions.

A replicate of this experiment using 4 of those 8 accessions (Alaska, NK Nemax, Pollen, Zephir) was performed in the next season. Vernalized plants were potted into containers on 07.04.2016 with 5 plants per container. The stress treatment was induced on 23.05.2016. At the same day, the plants were bagged to ensure self-pollination. The resulting seeds were harvested on 23.08.2016 and stored under dry conditions.

### Analysis of seed quality

Seed oil content, seed protein content and seed glucosinolate content was determined using near-infrared reflectance spectroscopy (NIRS, Unity SpectraStar 2500, Brookfield, USA). NIRS measurements were performed using standard procedures as described in [32].

Fatty acid quantification was performed by gas chromatography (GC) analysis. In a test tube, 300 mg seeds were grinded together with 2 mL petroleum benzine for 2 min at maximum speed with a T25 digital Ultra Turrax sample grinder (IKA Works, Inc. Wilmington, USA). Another 2 mL petroleum benzine were added and the sample was vortexed for 10 s. After 30 min, 0.8 mL of supernatant was pipetted into a new test tube, which was placed under the laboratory fume hood until the petroleum benzine was completely evaporated. Subsequently, 2 mL sodium methylate were added to the remaining oil in the test tube and the sample was vortexed for 10 s. The sample was covered and left to settle for 30 min. Afterwards 1.7 mL iso-octane were added and the solution was shaken carefully. The sample was again covered and left to settle for 30 min. Supernatant of the upper phase was collected and used for GC analysis (TRACE GC Ultra Gas Chromatograph, Thermo Scientific, Waltham, USA). GC analysis was performed with the GC capillary column BPX70 (SGE Analytical Science, Milton Keynes, GB). As specified for rapeseed oil fatty acid composition, the fatty acid methyl ester mixture F07 (Carl Roth GmbH + Co. KG, Karlsruhe, Deutschland) was used as a standard. For analysis, the following temperature ramps were used: Start at 160 °C for 1 min, incremental increase of temperature every min by 15 °C until temperature reaches 210 °C, 210 °C for 30 s, 220 °C for 6 min. The samples were measured in two technical replicates.

### Determination of seed germination characteristics

Mean germination time (MGT; in h), germination rate within 96 h (GR96; in %) and uniformity of germination, measured as the difference between the time to reach 10 and 90% of germination (U; in h), were phenotyped under in vitro conditions at 20 °C, using the automated phenotyping platform of the variety control office of the French national seed testing agency (Station Nationale d’Essais de Semences, Groupe d’Etude et de contrôle des Variétés et des Semences—GEVES, Angers, France). GR96 can be considered as the absolute germination rate, as no further increase in germination was observed 96 h after imbibition start. Germination was assayed under well-watered conditions as well as under moderate osmotic stress at an intensity of − 0.5 MPa. Seed germination analysis was carried out with 4 × 25 seeds per genotype and treatment. Detailed information about the phenotyping system is given by [33].

### Analysis of seedling vigour

For analysis of seedling vigour, 12 seeds harvested from each container were sown in small plastic pots (8 × 7 × 7 cm) filled with 350 g sand. Germination and seedling growth took place in the greenhouse under controlled conditions as follows: 12 h/12 h light/dark, temperature 20–24 °C during the light period and 12 °C - 16 °C during the dark period; minimum light intensity 10 klx during the light period. The pots were watered via dish watering. Water capacity of the sand was determined as the difference in weight between sand filled pots dried for 24 h at 85 °C in a drying cabinet, and sand filled pots after watering. Water capacity was subsequently maintained up at 75% during the whole experiment. Seedlings were harvested 7 d after sowing. In each case, 12 seedlings were pooled and samples were shock-frozen immediately after fresh weight (FW) determination of each pooled sample. Consequently, three pooled samples were analyzed for each accession and maternal treatment. The shock-frozen samples were freeze-dried for 5 d and used for subsequent metabolite analysis.

### Metabolite analysis

Metabolite analysis was performed on a Waters Acquity ultraperformance liquid chromatography machine with diode array detection (UPLC-DAD) using methods and software described in the Waters Corporation user manual. The manual was adapted for oilseed rape tissue by [34, 35]. The AccQtag method was used to quantify amino acids and the integration software Empower (Waters Corporation, Milford, USA) was used for analysis. Samples were resuspended in 100 mL distilled water. Subsequently, 5 mL were derivatized using AccQTag Ultra Derivatization Kit, according to the manufacturer’s recommendations. An external standard of 100 mmol/L of each amino acid was run every 10 samples. Quantification of sugars was performed using a gas chromatography-flame ionization detector (GC-FID) System from Agilent Technologies (Santa Clara, CA, USA) according to [36]. The integrated Agilent software ChemStation Rev.B.04.02 was used for data analysis. Samples were resuspended in 50 mL pyridine (100%) with methoxamine hydrochloride (240 mmol/L), then derivatized with 50 mL MSTFA (N-methyl-N-(trimethylsilyl)trifluoro acetamide) (100%). An external standard containing 400 mmol/L of each sugar, sugar alcohol and organic acid was run every 10 samples.

### Statistical analysis

Data analysis was carried out using R (version 3.3.3, 2017). Compliance with normal distribution and homogeneity of variances were evaluated using a Bartlett test. After global testing with ANOVA, Student’s *t*-test was applied for pairwise comparisons. In terms of consistency testing, no adjustment was chosen for the statistical analysis. Significances were tested at levels of *p* < 0.1′, *p* < 0.05*, *p* < 0.01** and *p* < 0.001***. Correlations were calculated applying the Pearson’s product-moment correlation.

## Additional files

Additional file 1: Seed fatty acid composition: Relative amounts of fatty acids in the seeds of 8 diverse winter oilseed rape genotypes cultivated under control and drought stress conditions. Values are means of three replicates + standard errors. Significant differences at *p* < 0.1′, *p* < 0.05*, *p* < 0.01** and *p* < 0.001***. (XLSX 13 kb)

Additional file 2: Effect of maternal drought stress on seedling fresh weight of 4 winter oilseed rape genotypes cultivated in a semi-controlled container trial after self-pollination. Bars are means of three replicates with standard errors. Significant differences at p < 0.1′. (TIF 4218 kb)

Additional file 3: Seedling metabolome: Concentrations of different nitrogen compounds, amino acids, sugars and organic acids in seedlings derived from 8 diverse winter oilseed rape genotypes cultivated under control and drought stress conditions. Values are means of three replicates + standard errors. Significant differences at *p* < 0.1′, *p* < 0.05*, *p* < 0.01** and *p* < 0.001***. (XLSX 18 kb)

Additional file 4: Trait correlations: Table summarizing all significant correlations (*p* < 0.1) between the two main parameters total maternal seed yield (Yield) and seedling fresh weight building of the progeny (SFW) and different single yield, seedling growth, seed quality and seedling metabolome parameters, determined in 8 diverse winter oilseed rape genotypes grown in a semi-controlled container trial under control (C) and drought stress conditions (S). S + C: Correlations were calculated among both treatments. S/C: Correlations were calculated between the relative values of each trait-trait combinations as quotient of value from stress treatment to value from control treatment. Seeds/Sil.: Number of seeds per silique, Seeds/Pl.: Number of seeds per plant. (JPG 236 kb)

Additional file 5: Soil water content during the stress trial before watering (above) and after watering (below). The numbers indicate days after stress initiation. Values are means of three replicates + standard errors. Significant differences at p < 0.1′, p < 0.05*, p < 0.01** and p < 0.001***. (TIF 4218 kb)

## Abbreviations

++: high seed erucic acid and glucosinolate content
Arg: Arginine
Asn: Asparagine
Asp: Aspartate
BBCH: Scale for evalutaion of plant developmental stage developed from Biologische Bundesanstalt, Bundessortenamt und Chemische Industrie
Cys: Cysteine
FAE1: Fatty acid elongase 1
FW: Fresh weight
GABA: γ-aminobutyric acid
Gln: Glutamine
Glu: Glutamate
Gly: Glycine
GR96: Germination rate within 96 h after imbibition start
His: Histidine
Hyp: Hydroxyproline
Ile: Isoleucine
Leu: Leucine
Lys: Lysine
Met: Methionine
Met.-Cys: Methyl-cysteine
MGT: Mean germination time
Orn: Ornithine
PCA: Principal component analysis
Phe: Phenylalanine
Pro: Proline
Ser: Serine
SMCSO: S-methylcystein sulfoxide
Thr: Threonine
Trp: Tryptophane
Tyr: Tyrosine
U: Uniformity
Val: Valine
α-Ala: α-Alanine
β-Ala: β-Alanine

## References

[1] Bates BC, Kundzewicz ZW, Wu S, Palutikof JP, Eds. Climate change and water. Technical Paper of the Intergovernmental Panel on Climate Change, IPCC Secretariat, Geneva. 2008. 210 pp. https://drive.google.com/file/d/0B1gFp6Ioo3akcFFFeGRRVFNYM0E/view.

[2] Li Y, Ye W, Wang M, Yan X (2009). Climate change and drought. A risk assessment of crop-yield impacts. Clim Res.

[3] FAOSTAT Food and agriculture data. Food and Agriculture Organisation of the United Nations, Rome. 2017. http://www.fao.org/faostat/en. Accessed 8 Jan 2017.

[4] Wittkop B, Snowdon RJ, Friedt W (2009). Status and perspectives of breeding for enhanced yield and quality of oilseed crops for Europe. Euphytica.

[5] Jensen CR, Mogensen VO, Mortensen G, Fieldsend JK, Milford GFJ, Andersen MN (1996). Seed glucosinolate, oil and protein contents of field-grown rape (*Brassica napus* L.) affected by soil drying and evaporative demand. Field Crop Res.

[6] Gao J, Thelen KD, Min DH, Smith S, Hao X, Gehl R (2010). Effects of manure and fertilizer applications on canola oil content and fatty acid composition. Agron J.

[7] Onemli F (2014). Fatty acid content of seed at different development stages in canola on different soil types with low organic matter. Plant Prod Sci.

[8] Delourme R, Falentin C, Huteau V, Clouet V, Horvais R, Gandon B (2006). Genetic control of oil content in oilseed rape (*Brassica napus* L.). Theor Appl Genet.

[9] Ellis RH (1992). Seed and seedling vigour in relation to crop growth and yield. Plant Growth Regul.

[10] TeKrony DM, Egli DB (1991). Relationship of seed vigor to crop yield: a review. Crop Sci.

[11] Hohmann M, Stahl A, Rudloff J, Wittkop B, Snowdon RJ (2016). Not a load of rubbish. Simulated field trials in large-scale containers. Plant Cell Environ.

[12] Richards RA, Thurling N (1978). Variation between and within species of rapeseed (*Brassica campestris* and *B. napus*) in response to drought stress. I sensitivity at different stages of development. Aust J Agric Res.

[13] Champolivier L, Merrien A (1996). Effects of water stress applied at different growth stages to *Brassica napus* L. var. *oleifera* on yield, yield components and seed quality. Eur J Agron.

[14] Din J, Khan SU, Ali I, Gurmani AR (2011). Physiological and agronomic response of canola varieties to drought stress. J Anim Plant Sci.

[15] Hatzig SV, Frisch M, Breuer F, Nesi N, Ducournau S, Wagner MH (2015). Genome-wide association mapping unravels the genetic control of seed germination and vigor in *Brassica napus*. Front Plant Sci.

[16] Dorion S, Lalonde S, Saini HS (1996). lnduction of male sterility in wheat by meiotic-stage water deficit is preceded by a decline in invertase activity and changes in carbohydrate metabolism in anthers. Plant Physiol.

[17] Saini HS (1997). Effects of water stress on male gametophyte development in plants. Sex Plant Reprod.

[18] Meyer RC, Törjék O, Becher M, Altmann T (2004). Heterosis of biomass production in *Arabidopsis*. Establishment during early development. Plant Physiol.

[19] Bouchereau A, Clossais-Besnard N, Bensaoud A, Leport L, Renard M (1996). Water stress effects on rapeseed quality. Eur J Agron.

[20] Aslam MN, Nelson MN, Kailis SG, Bayliss KL, Speijers J, Cowling WA (2009). Canola oil increases in polyunsaturated fatty acids and decreases in oleic acid in drought-stressed Mediterranean-type environments. Plant Breed.

[21] Guo Y, Si P, Wang N, Wen J, Yi B, Ma C, et al. Genetic effects and genotype × environment interactions govern seed oil content in *Brassica napus* L. BMC Genet. 2017;18(1). 10.1186/s12863-016-0468-0. 10.1186/s12863-016-0468-0 PMC5217400 28056775

[22] Baud S, Lepiniec L (2009). Regulation of *de novo* fatty acid synthesis in maturing oilseeds of *Arabidopsis*. Plant Physiol Bioch.

[23] Lämke J, Bäurle I (2017). Epigenetic and chromatin-based mechanisms in environmental stress adaptation and stress memory in plants. Genome Biol.

[24] Boyko A, Blevins T, Yao Y, Golubov A, Bilichak A, Ilnytskyy Y (2010). Transgenerational adaptation of *Arabidopsis* to stress requires DNA methylation and the function of dicer-like proteins. PLoS One.

[25] Ou X, Zhang Y, Xu C, Lin X, Zang Q, Zhuang T (2012). Transgenerational inheritance of modified DNA methylation patterns and enhanced tolerance induced by heavy metal stress in rice (*Oryza sativa* L.). PLoS One.

[26] Lang-Mladek C, Popova O, Kiok K, Berlinger M, Rakic B, Aufsatz W (2010). Transgenerational inheritance and resetting of stress-induced loss of epigenetic gene silencing in *Arabidopsis*. Mol Plant.

[27] Whittle CA, Otto SP, Johnston MO, Krochko JE (2009). Adaptive epigenetic memory of ancestral temperature regime in *Arabidopsis thaliana*. Botany.

[28] Lacey EP (1995). Parental effects in *Plantago lanceolata* L. I.: a growth chamber experiment to examine pre- and postzygotic temperature effects. Evolution.

[29] Galloway LF (2001). The effect of maternal and paternal environments on seed characters in the herbaceous plant *Campanula americana* (*Campanulaceae*). Am J Bot.

[30] Suter L, Widmer A (2013). Environmental heat and salt stress induce transgenerational phenotypic changes in *Arabidopsis thaliana*. PLoS One.

[31] Bus A, Körber N, Snowdon RJ, Stich B (2011). Patterns of molecular variation in a species-wide germplasm set of *Brassica napus*. Theor Appl Genet.

[32] Tillmann P, Reinhardt TC, Paul C (2000). Networking of near infrared spectroscopy instruments for rapeseed analysis: a comparison of different procedures. J Near Infrared Spectrosc.

[33] Demilly D, Ducournau S, Wagner MH, Dürr C, Dutta Gupta S, Ibaraki Y (2014). Digital imaging of seed germination. Plant image analysis - fundamentals and applications.

[34] Albert B, Le Cahérec F, Niogret MF, Faes P, Avice JC, Leport L (2012). Nitrogen availability impacts oilseed rape (*Brassica napu*s L.) plant water status and proline production efficiency under water-limited conditions. Planta.

[35] Deleu C, Faes P, Niogret MF, Bouchereau A (2013). Effects of the inhibitor of the g-aminobutyrate-transaminase, vinyl-gaminobutyrate, on development and nitrogen metabolism in *Brassica napus* seedlings. Plant Physiol Biochem.

[36] Lugan R, Niogret MF, Kervazo L, Larher FR, Kopka J, Bouchereau A (2009). Metabolome and water status phenotyping of *Arabidopsis* under abiotic stress cues reveals new insight into ESK1 function. Plant Cell Environ.

