# Supplementary figures and images for: Drought stress has transgenerational effects on seeds and seedlings in winter oilseed rape (Brassica napus L.)
Source: BMC Plant Biol. 2018 Nov 23;18:297. doi: 10.1186/s12870-018-1531-y (PMC6251133; doi:10.1186/s12870-018-1531-y)

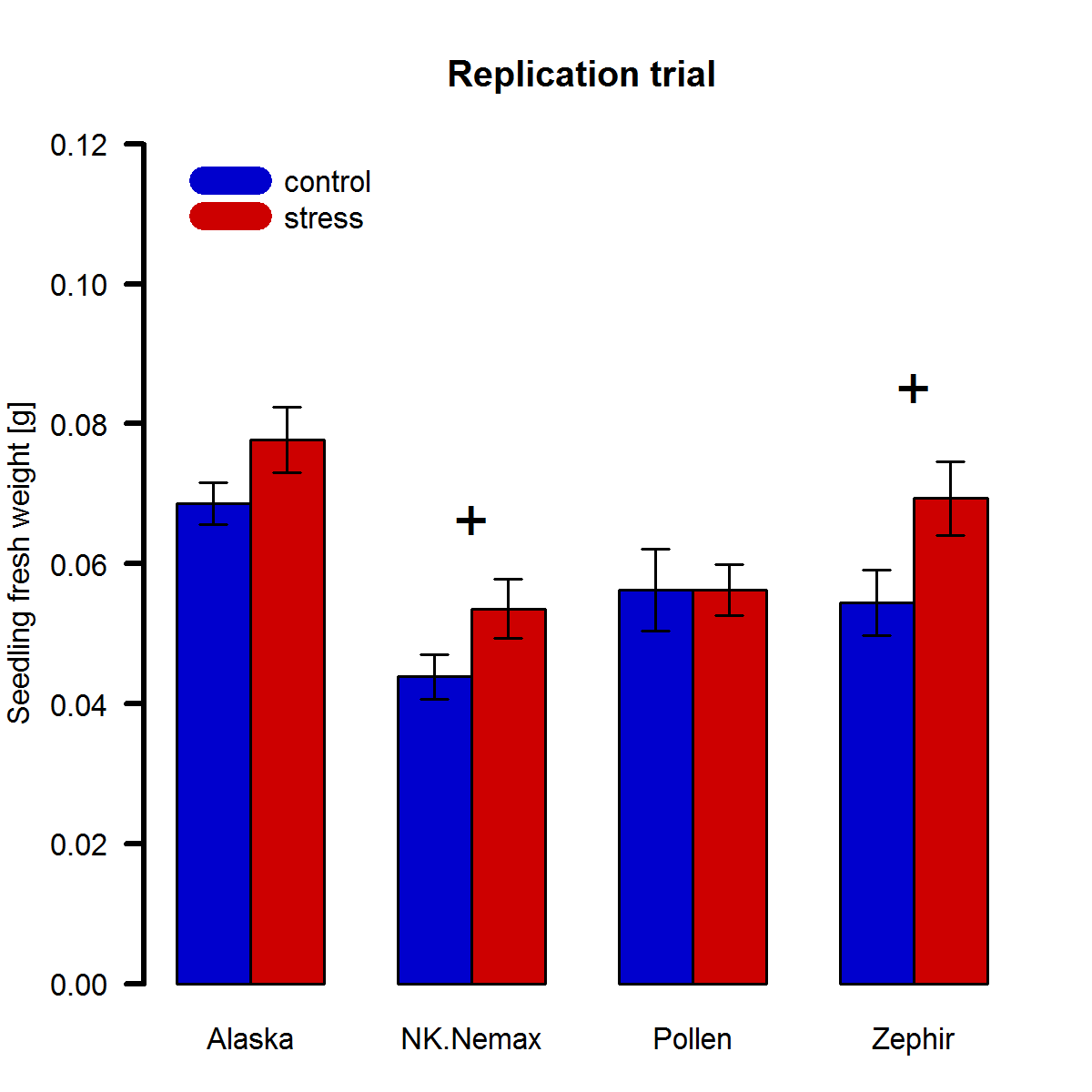

Supplement: Supplementary file 2 — Effect of maternal drought stress on seedling fresh weight of 4 winter oilseed rape genotypes cultivated in a semi-controlled container trial after self-pollination. Bars are means of three replicates with standard errors. Significant differences at p < 0.1′. (TIF 4218 kb) [file 12870_2018_1531_MOESM2_ESM.tif]

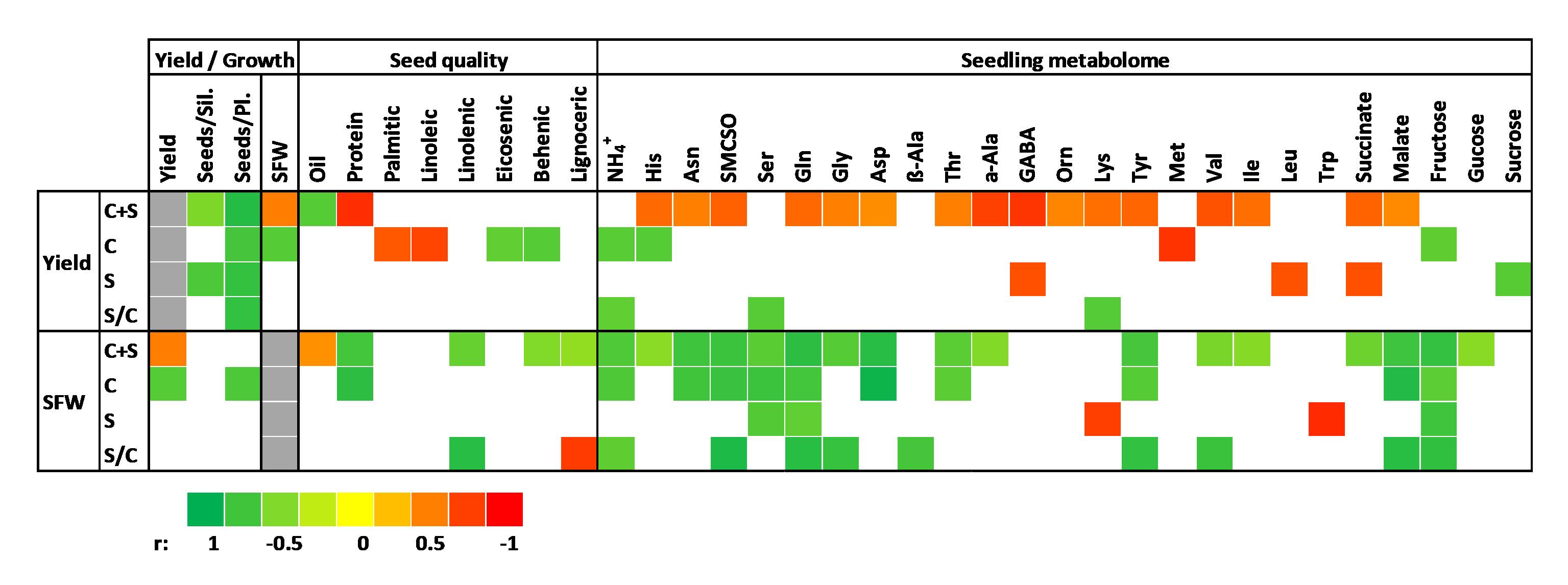

Supplement: Supplementary file 4 — Trait correlations: Table summarizing all significant correlations (p < 0.1) between the two main parameters total maternal seed yield (Yield) and seedling fresh weight building of the progeny (SFW) and different single yield, seedling growth, seed quality and seedling metabolome parameters, determined in 8 diverse winter oilseed rape genotypes grown in a semi-controlled container trial under control (C) and drought stress conditions (S). S + C: Correlations were calculated among both treatments. S/C: Correlations were calculated between the relative values of each trait-trait combinations as quotient of value from stress treatment to value from control treatment. Seeds/Sil.: Number of seeds per silique, Seeds/Pl.: Number of seeds per plant. (JPG 236 kb) [file 12870_2018_1531_MOESM4_ESM.jpg]

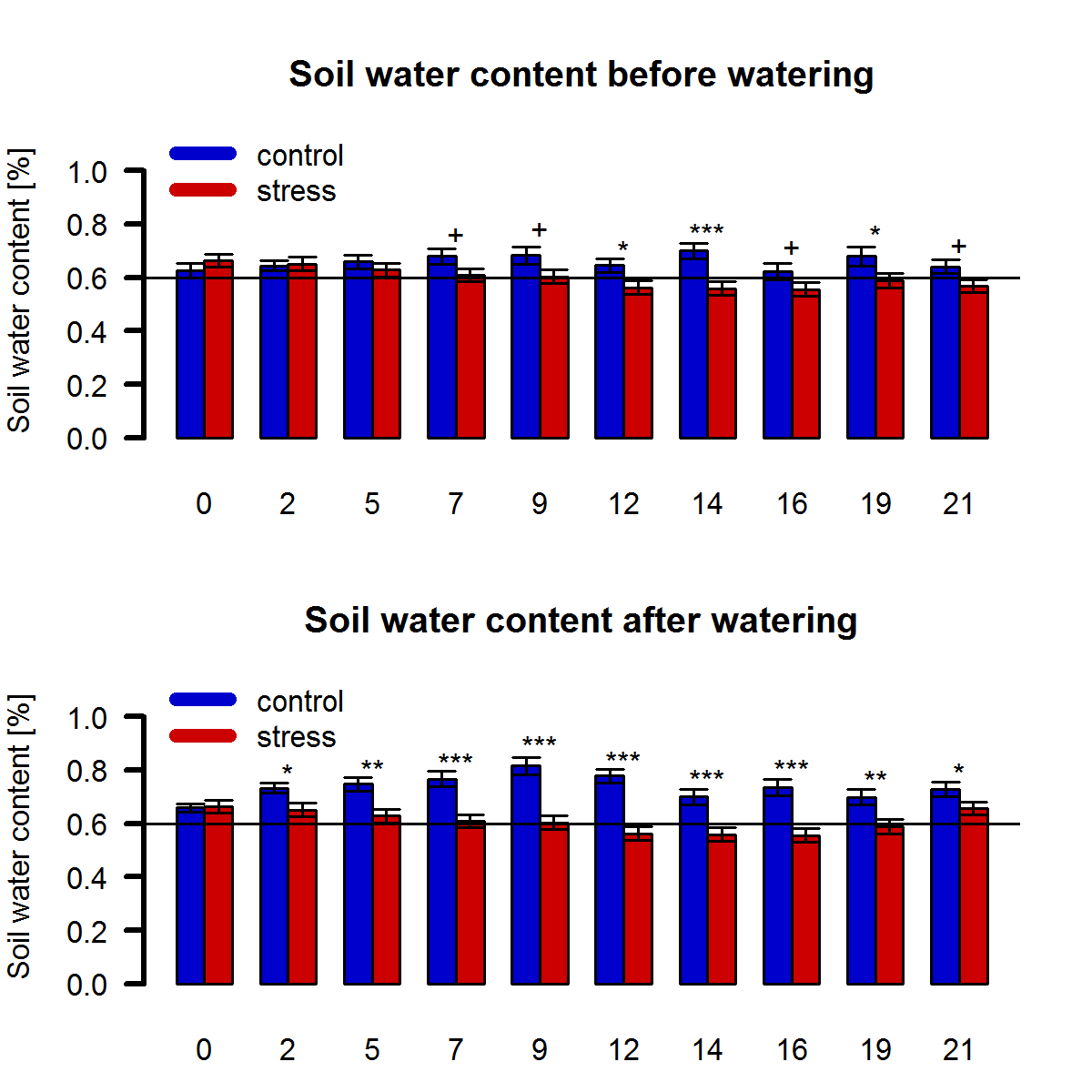

Supplement: Supplementary file 5 — Soil water content during the stress trial before watering (above) and after watering (below). The numbers indicate days after stress initiation. Values are means of three replicates + standard errors. Significant differences at p < 0.1′, p < 0.05*, p < 0.01** and p < 0.001***. (TIF 4218 kb) [file 12870_2018_1531_MOESM5_ESM.tif]
